# Early identification in primary health care of people at risk for sick leave due to work-related stress – study protocol of a randomized controlled trial (RCT)

**DOI:** 10.1186/s12889-016-3852-9

**Published:** 2016-11-25

**Authors:** Kristina Holmgren, Christine Sandheimer, Ann-Charlotte Mårdby, Maria E. H. Larsson, Ute Bültmann, Dominique Hange, Gunnel Hensing

**Affiliations:** 1 Department of Health and Rehabilitation, Institute of Neuroscience and Physiology, The Sahlgrenska Academy at the University of Gothenburg, Gothenburg, Sweden; 2 Section for Epidemiology and Social Medicine, Institute of Medicine, the Sahlgrenska Academy at the University of Gothenburg, Gothenburg, Sweden; 3 The Sahlgrenska University Hospital, Gothenburg, Sweden; 4 Novo Nordisk A/S, Box 50587, SE-202 15 Malmö, Sweden; 5 Närhälsan Research and Development, Primary Health Care, Region Västra Götaland, Sweden; 6 Department of Health Sciences, Community and Occupational Medicine University of Groningen, University Medical Center Groningen, Groningen, The Netherlands; 7 The unit of Primary Health Care, Institute of Medicine, The Sahlgrenska Academy at the University of Gothenburg, Gothenburg, Sweden

**Keywords:** Psychosocial work factors, Work Stress Questionnaire (WSQ), Intervention, Organizational climate, Work commitment

## Abstract

**Background:**

Early identification of persons at risk of sickness absence due to work-related stress is a crucial problem for society in general, and primary health care in particular. Tho date, no established method to do this exists. This project’s aim is to evaluate whether systematic early identification of work-related stress can prevent sickness absence. This paper presents the study design, procedure and outcome measurements, as well as allocation and baseline characteristics of the study population.

**Method/design:**

The study is a two-armed randomized controlled trial with follow-up at 3, 6 and 12 months. Non-sick-listed employed women and men, aged 18 to 64 years, who had mental and physical health complaints and sought care at primary health care centers (PHCC) were eligible to participate. At baseline work-related stress was measured by the Work Stress Questionnaire (WSQ), combined with feedback at consultation, at PHCC. The preventive intervention included early identification of work-related stress by the WSQ, GP training in the use of WSQ, GP feedback at consultation and finding suitable preventive measures. A process evaluation was used to explore how to facilitate future implementation and structural use of the WSQ at the PHCC. The primary outcome to compare the preventive sick leave intervention by the general practitioner (GP) versus treatment as usual is sick leave data obtained from the Swedish Social Insurance Agency register.

**Discussion:**

Early screening for sick leave due to work-related stress makes it possible not only to identify those at risk for sick leave, but also to put focus on the patient’s specific work-related stress problems, which can be helpful in finding suitable preventive measures. This study investigates if use of the WSQ by GPs at PHCCs, combined with feedback at consultation, prevents future sickness absence.

**Trial registration:**

ClinicalTrials.gov. Identifier: NCT02480855. Registered 20 May 2015

## Background

Work-related stress is common in many European countries, with Sweden representing the highest level of reported work stress in Europe [1, 2]. A number of organizational and psychosocial work-related factors are found to be associated with stress, which in turn might result in adverse health effects and illness, and a higher risk of sick leave. Work-related factors, such as poor organizational climate, in terms of intolerance at work [3, 4], conflicts [5, 6], and injustice at work [7] are associated with stress, poor health and subsequent sick leave. Being engaged in work or committed to work is basically considered to have a positive influence on both the individuals’ well-being and that of the organization [8, 9]. It has been demonstrated, though, that being too engaged, or over-committed, is a risk factor for sickness presenteeism [10], work-related stress [11] and poor health [12]. These organizational and psychosocial working life stressors and strains affect people negatively and result in various mental and physical health complaints, even prior to sick-listing [13–15]. People with these complaints often consult their primary health care physician [16–18] long before they even contemplate taking sick leave [11, 19]. It may well be that neither the patient, nor the general practitioner (GP) is aware that their symptoms could be caused by organizational and psychosocial factors at work. Because many patients might be at risk of disability and long-term sick leave, it is of immense value to identify these persons early and to take preventive actions [20].

Providing sickness certificates is a common task for GPs in Sweden [21, 22]. One third of Swedish GPs reported having 1–5 consultations each per week concerning sick leave [21]. This indicates that they often deal with assessing level of patients’ work incapacity in their everyday practice [21, 23]. GPs often found the decision about issuing a sickness certificate difficult, especially if the patients describe symptoms without clinical findings [21, 24]. Likewise, GPs stated that they had poor knowledge of the workplace environment and the labor market [23, 25], and they reported that they barely talked to patients about their work situation [26, 27]. Today, GPs have no established practice for early identification of patients at risk for sick leave caused by adverse psychosocial factors.

The Work Stress Questionnaire (WSQ) has been designed specifically for early identification of people at risk for sick leave due to work-related stress, and was developed in the context of primary health care [11, 19, 28]. The WSQ is based on the idea that personal characteristics and environmental factors are interdependent, and that changes in either of these influences the possibilities for a sustainable work performance [29–32]. Experiences from sick-listed people [11] contributed to the questionnaire development, and showed that a poor organizational climate, as exemplified by indistinct leadership and conflicts at work, in combination with high work commitment, such as excessive individual demands and responsibility, was crucial for future sick-listing [11]. A prospective Swedish primary health care study [19] found that high stress due to poor organizational climate at baseline, measured with the WSQ, more than doubled the risk for sick leave at follow-up. Combined with high stress due to high work commitment the risk for sick leave increased fourfold.

Early screening makes it possible not only to identify patients at risk for sick leave but also to identify the patient’s specific problems, which makes for the use of preventive measures and efficient treatment [33]. During the patient–GP consultation, tailored preventive measures for work-related stress can be suggested that might lower the risk of future sick leave. Since the WSQ takes both work-related factors and personal characteristics into account, it is possible to identify work-related stress from both an environmental and a personal perspective. Thus, the WSQ gives the GP the opportunity to direct preventive measures towards either the person or the workplace, or both. Therefore, it is important in GP practice to identify the patient’s specific problems at work early, to communicate them to the patient, and to recommend suitable preventive measures.

### Aims and hypothesis

The overall aim of this randomized controlled trial (RCT) is to evaluate whether systematic use of the WSQ, combined with feedback at consultation, can serve as a method for health care professionals in primary health care centers (PHCCs) to prevent or reduce sick leave due to work-related stress during a 12-month follow-up period. The preventive intervention will be compared versus treatment as usual (TAU). The aim is also to evaluate whether there are differences between the intervention group and the control group in healthcare measures and the prescribed medications at follow-up. In a process evaluation, the systematic use of the WSQ combined with feedback at consultation is examined.

The hypothesis of this RCT is that patients who answer the WSQ, when combined with feedback at GP consultation, will have fewer sick leave days during the year after intervention compared with those who receive TAU.

This paper presents the study design, the procedure, the outcome measurements, the allocation and the baseline characteristics of the study population. The project is still ongoing, with follow-up data to be collected and analyzes to be done. The RCT was designed in accordance with CONSORT recommendations [34].

## Method and design

### Study context

In Sweden, the social insurance scheme provides benefits to people who cannot work because of disease or injury. Those gainfully employed are covered for the first 14 days (except for one qualification day) by their employer, and after that period benefits are granted from the Social Insurance Agency. From day 8, a medical certificate is required. Providing sickness certificates is a common task for GPs in Sweden [21, 22]. This study is conducted in PHCCs in the Västra Götaland region with a population of 1.6 million inhabitants, around 17% of the Swedish population. The region has approximately 200 public and private PHCCs with approximately 800 employed GPs.

This RCT study is part of the TIDAS project within the New Ways research program at the Section for Epidemiology and Social Medicine, Institute of Medicine, Sahlgrenska Academy, University of Gothenburg.

### Study design and recruitment

This study was designed as a two-armed RCT for early identification of people at risk for sick leave due to work-related stress consulting PHCCs. The recruitment of PHCCs took place from May 2015 to November 2015. Out of the Västra Götaland region’s 200 PHCCs, 51 public and private PHCCs located in rural and urban areas in and around Gothenburg were identified and consecutively invited to participate. In all, seven PHCCs (four public and three private) participated. The PHCCs were economically compensated for each participant recruited.

### Randomization

GPs and residents who worked in the clinic at participating PHCCs at least 50% of the time were randomized to either the intervention or the control group. The names of all GPs at the participating PHCC were written on slips of paper that were folded and then mixed in a non-transparent bowl. Colleagues that were not involved in the RCT drew the names one at a time, and the names were alternately included in the intervention or the control group.

### Procedure

Prior to the intervention period, the research team visited the participating PHCC and presented the study procedure. The control GPs were instructed to carry on as usual with their consultations. The intervention GPs received a brief training for the intervention, which included knowledge on the relationship between psychosocial factors at work, stress, health and sickness absence. GPs also received instructions on how to use, operationalize and interpret the WSQ, and on how to give feedback to the participants and refer patients at risk. Both oral and written information on the services of the primary health care specialists and occupational healthcare was presented to the GPs.

### Masking (blinding)

Neither participants nor the GP were blinded to allocation in the RCT because of the nature of the intervention. All participants were given information on the study and signed consent forms before the patient–GP consultations. However, the control GPs were not informed when a patient for consultation was a study participant, and the controls filled in the questionnaires after consultation.

### Eligibility to participate

#### Inclusion criteria

Non-sick-listed employed women and men aged 18 to 64 years who saw a GP at the PHCCs in the Västra Götaland region for mental and/or physical health complaints, including depression, anxiety, musculoskeletal disorders, gastrointestinal, cardiovascular symptoms and other stress-related symptoms were invited [16–18].

#### Exclusion criteria

Patients seeking care for diabetes, urinary tract infections, infections, chronic obstructive lung disease, fractures, lump and spots, allergy and psychiatric diagnoses such as schizophrenia, other psychoses or bipolar diagnoses, as well as medical check-ups were excluded. Pregnant women were also excluded because they might be at risk for pregnancy-related sick leave during the follow-up period. Patients currently on sick leave and those who had been off work for a total of 7 days or more during the last month because of sickness, with or without medical record, were excluded, as well as those with a full or part-time disability pension.

### Sample size

A power calculation was performed to determine the number of participants needed to detect at least a 15% [35] difference between the intervention group and the control group concerning the primary outcome, i.e. the number of registered sick leave days (i.e. >14 days or more) during 12 months after inclusion. With a two-sided test, statistical significance of *p* < 0.05 and 80% power, at least 135 participants were needed in each group.

### Data collection

**Fig. 1:**
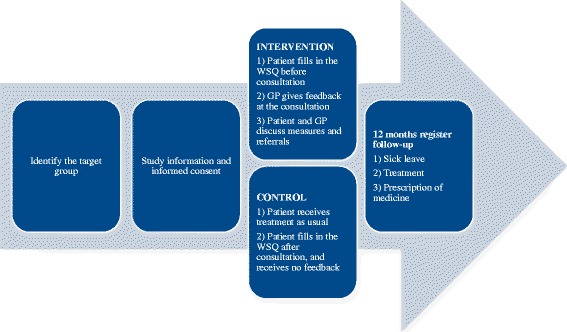
The procedure of datacollection and follow-ups, TIDAS in New Ways

Data collection took place over a period of 4–8 weeks per center (except for one center, where the data collection took 12 weeks) from May 2015 until January 2016. During data collection, a research assistant was stationed at the PHCC. The research assistant identified and recruited the eligible participants and gave oral and written information on the study. All participants were also asked to provide informed consent for the study, including linking records to registers during follow-up (Fig. 1).

### Intervention group

The intervention consisted of the following components: GPs’ brief training in the use of the WSQ, participants’ completion of the WSQ, GP feedback at consultation and finding suitable preventive measures. The WSQ consists of 21 main questions grouped into four categories [28]. Two of the categories pertain to perceived stress due to *indistinct organization and conflicts* and perceived stress due to *individual demands and commitment*. Each of these two categories contains seven items. Response options are given on a four-point ordinal scale: ‘Not at all stressful’, ‘Less stressful’, ‘Stressful’, and ‘Very stressful’. The other two categories pertain to *influence at work* and *work interference with leisure time,* and contain four and three items, respectively, with response options given on a four-point ordinal scale: ‘Yes, always’, ‘Yes, rather often’, ‘No, seldom’ and ‘No, never’. The reliability and face validity of the WSQ has been tested and found to be good [28].

Before the GP consultation, each participant filled in the WSQ and questions on background characteristics, which took around 15 min. The research assistant computed the WSQ and handed over the result to the GP before consultation. At the patient–GP consultation, the GPs were instructed to give feedback to the participant by communicating the results of the WSQ, and discussing possible measures, such as referrals to PHCC’s specialists or to the participant’s occupational healthcare (Fig. 1).

Directly after each patient–GP consultation, the GP filled in a questionnaire concerning their adherence to the instructions.

### Control group

Control participants received TAU, i.e. an ordinary patient–GP consultation. The GP had no information on whether the patient was a study participant. After the GP consultation, the participant completed the WSQ and answered questions on background characteristics (Fig. 1).

### Baseline assessments

Self-reported baseline characteristics were collected by questionnaire on gender (female, male), age (years), country of birth (Nordic, other), educational level (compulsory schooling, secondary school education, university or higher education), occupation, employer (private, public, self-employed), employment status (permanent, temporary, self-employed), and the reason for consulting the PHCC (mental and/or physical health complaints).

### Follow-up outcome measurements

All registered data will be collected one year after last inclusion, i.e. January 2017.

#### Primary outcome

The number of registered sick leave days (i.e. 14 days or more) and number of absence periods during the 12 months after inclusion covered by sickness benefit will be obtained from the Swedish social insurance agency’s Micro Database for Analyzing Social insurance (MiDAS) as well as data on full- and part-time sick leave and sickness and activity compensation.

#### Secondary outcome measurements

Short term sick leave (<14 days) and present work status are collected at 3, 6, and 12 months by telephone or email follow-up.

Healthcare measures will be obtained from the Vega database, which covers data on hospital and primary health care patients in the Västra Götaland region of Sweden. Data concerning diagnoses, number of visits, referrals, and content of consultations and measures during the 12 months following inclusion.

Data on prescribed medications will be obtained from the Swedish Prescribed Drug Register, a national population-based register established in 2005, which contains information on all purchases of prescribed medications in pharmacies [36]. Data concerning the name and amount of purchased medication, date dispensed, and dosage instructions during the 12 months following inclusion.

### Statistical analysis

The analyses will follow the intention-to-treat principle [37]. Per protocol analyses will be conducted to examine if deviations from the protocol have caused bias. Both descriptive and analytic statistics will be used to compare the intervention group and the control group. Analysis will be adjusted for gender and other possible confounders. Sub-group analyses will be done with regard to gender, and if possible given the number of participants, age and diagnostic groups. Non-parametric statistics will be used when ordinal data are analyzed. Otherwise, parametric statistics will be used [37].

### Time plan of the RCT

The enrollment of PHCCs took place from May 2015 to November 2015. The intervention took place between May 2015 and January 2016. Follow-up of sickness absence, healthcare measures and prescribed medications in the registers will be completed one year after last inclusion, i.e. January 2017. Short-term sick leave is followed-up by telephone or e-mail at 3, 6, and 12 months until January 2017.

### Process evaluation, design and procedure

Both qualitative and quantitative methods were used in the process evaluation. The target group of the process evaluation consisted of the intervention GPs. The GPs’ considerations on management before, during and after intervention were assessed by questionnaires. Prior to the brief training, the GPs answered questions on readiness to use the WSQ in patient–GP consultation. Directly after each patient–GP consultation, the GP answered questions on adherence to the study protocol. After data collection, the GP answered questions on the feasibility of using the WSQ in patient–GP consultation in daily practice in the future.

After the baseline data collection was completed at each PHCC, all intervention GPs at that particular PHCC were invited to focus group discussions that explored the GPs’ perception of the systematic use of the WSQ. Oral and written information was given and informed consent for the focus group study was provided. The group sessions were held at the PHCC and were moderated by a researcher experienced in focus group methodology. The discussions focused on the following key questions: views on the content of the intervention, how to improve the process, views on the readiness to use the WSQ combined with feedback in daily practice, and how to facilitate future implementation and permanent use of the WSQ at the PHCCs. The group sessions were audio taped, transcribed verbatim and analyzed according to the method of Krueger [38].

### Allocation and baseline characteristics

**Fig. 2:**
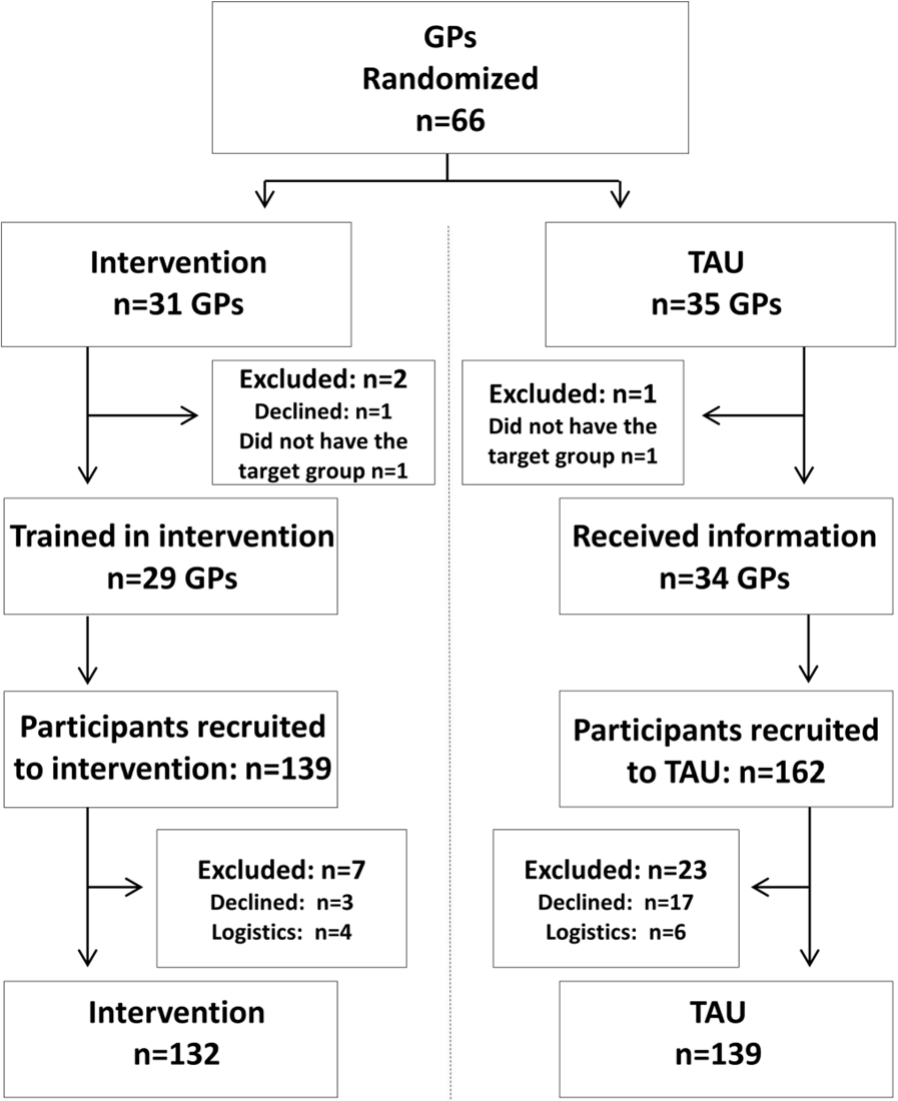
Flow chart of enrolment, allocation and baseline, TIDAS in New Ways

In total, 66 GPs were randomized to either the intervention group or to the control group. One GP declined participation and two GPs were excluded because of not having the target group at consultation. The intervention group (systematic use of the WSQ and feedback during patient–GP-consulting) consisted of *n* = 29 GPs and the control group (TAU) of *n* = 34 GPs (Fig. 2).

During the inclusion period, 301 non-sick-listed employed women and men aged 18 to 64 years who sought care at the seven participating PHCCs in the Västra Götaland region and fulfilled the inclusion criteria were asked to participate in the study. Of these, 20 eligible patients (7%) declined to participate. A total of 10 patients (3%) were excluded because they left the PHCC before being asked to fill in the questionnaires. No statistically significant differences in responses by participant age or gender were found. The final study population consisted of 271 participants (Fig. 2), of which 132 belonged to the intervention group and 139 to the control group.

The mean age was 46 years (standard deviation = 12) in the intervention group and 43 years (standard deviation = 11) in the control group, with a larger proportion in the age group 51–64 among intervention participants. Also, a larger proportion in the intervention group was consulting the PHCC for musculoskeletal reasons. Otherwise, there were no statistically significant differences between the groups in terms of baseline characteristics concerning sociodemographic factors and reasons for consulting the PHCC (Table 1).

**Table 1:** Characteristics of participants in the intervention and control groups, *n* = 271, TIDAS in New Ways

	Intervention *n* = 132	Control *n* = 139
	n^a^ (%)	n^a^ (%)
Gender		
Female	88 (67)	97 (70)
Age categories		
19–30 years	21 (16)	26 (19)
31–50 years	58 (44)	76 (54)
51–64 years	53 (40)	37 (27)^c^
Birthplace		
Nordic countries	122 (93)	125 (90)
Other	9 (7)	14 (10)
Educational level		
Compulsory schooling	13 (10)	15 (11)
Secondary school	61 (46)	59 (42)
University or higher	57 (44)	65 (47)
Occupational class		
Skilled/unskilled manual	49 (37)	58 (42)
Medium/low non-manual	60 (46)	56 (41)
High-level non-manual	23 (17)	24 (17)
Employer		
Private	61 (46)	68 (49)
Public	66 (50)	61 (44)
Self-employed	5 (4)	9 (7)
Reason for consultation^b^		
Mental or behavioral	75 (57)	69 (50)
Musculoskeletal	62 (47)	44 (32)^c^
Gastrointestinal	26 (20)	28 (20)
Cardiovascular	16 (12)	16 (16)
Other	29 (22)	27 (19)

^a^Dispersed numbers of participants are owing to internal

missing data

^b^Multiple responses were optional

^c^Statistically significant differences (tested with the 95% CI for difference in proportion)

## Discussion

There is a high level of sickness absence in Sweden, and stress-inducing factors at work play a large part in the sickness absence rate. Major efforts have been made to reduce sickness absence by restricting the sickness insurance scheme, by introducing monetary incentives to health care providers, and by specific recommended interventions, such as multimodal intervention and behavioral therapy [39]. However, none of these measures had long-term effects [39, 40]. Preventing and reducing sickness absence is challenging, and new measures are needed. Prolonged exposure to adverse psychosocial work conditions can cause stress, which in turn can lead to poor health. This scenario constitutes an obvious risk for people to be sick-listed [41–43]. People turn to their PHCCs to get help. The GPs, though, report little knowledge of work-related factors [21, 24], and rarely talk to their patients about organizational and psychosocial work-related factors [26, 27]. It is, however, essential to identify the patient at risk of being sick-listed at an early stage. This enables the GPs to take appropriate measures preventing health problems and subsequent sick leave. To date, no method exists that can be used in primary health care to identify people at risk for sick-listing due to work-related stress.

Up to now, many interventions have focused on treatment and rehabilitation of individuals already on sick leave. This is very important, but preventing sick leave is better still. Once a person is sick-listed, the return-to-work process is very costly, and this shows that much is to be gained from early identification. The focus of this project very much corresponds to needs expressed by individuals as well as society as a whole. This study is expected to show if early identification of work-related stress, using the WSQ, combined with feedback at consultation, can serve as a method for health care professionals in PHCCs to prevent or reduce sickness absence over a 12-month follow-up.

Fortunately, we reached our target sample size for participating patients. Also, the fact that few sociodemographic differences were identified between the groups was an advantage. The intervention participants were somewhat older and had a higher rate of musculoskeletal complaints as reasons for consultation. The rates of patients declining and being excluded from participation were low, and no differences concerning gender and age were observed. A limitation is that we did not collect data on non-participation patients’ reasons for consultation or reasons to decline participation because of ethical considerations.

The advantages of randomizing at the GP level were considered as twofold: the risk for variations in sociodemographic and socioeconomic factors between participating patients in intervention and controls were reduced, and engaging the whole PHCC to recruit both to intervention and control groups led to more participants attending in earlier studies [44]. The disadvantage of randomizing at the GP level was the risk for contamination, because the GPs might discuss the study procedure with each other. Because the inclusion period was short, and the intervention was brief and imbedded in ordinary daily practice, the contamination risk was considered rather low.

A strength of this project is that both qualitative and quantitative methods were used in the process evaluation. The focus group methodology involves group discussions and is distinguished from other qualitative group interviews by the explicit use of group interaction to collect data on a specific research topic. Communication between the participating focus group members is decisive for the outcome and the group process encourages the participants to clarify not only what they think, but also how and why they think in a certain way [38, 45]. An experienced group leader was chosen to moderate the sessions because the role of the group leader is essential in creating an open and friendly atmosphere that makes participants feel free to express their views [45]. In addition to using the questionnaires on GPs’ readiness and feasibility in the process evaluation, they will be analyzed in relation to the outcome variables.
